# Cannabis: What We Use, Why It Matters, and When It Is Prescribed (Ethics, Policy, and Practice)

**DOI:** 10.7759/cureus.89073

**Published:** 2025-07-30

**Authors:** Laresh N Mistry, Shreyas Neelkanthan, Saudamini More, Sumeet Agarwal, Himmat Jaiswal, Vivek Sharma

**Affiliations:** 1 Department of Pediatric and Preventive Dentistry, Bharati Vidyapeeth (Deemed to be University) Dental College and Hospital, Navi Mumbai, IND; 2 Department of Public Health Dentistry, Bharati Vidyapeeth (Deemed to be University) Dental College and Hospital, Navi Mumbai, IND; 3 Department of Prosthodontics, Bharati Vidyapeeth (Deemed to be University) Dental College and Hospital, Navi Mumbai, IND; 4 Department of Conservative Dentistry and Endodontics, Bharati Vidyapeeth (Deemed to be University) Dental College and Hospital, Navi Mumbai, IND; 5 Department of Periodontology, Bharati Vidyapeeth (Deemed to be University) Dental College and Hospital, Navi Mumbai, IND

**Keywords:** cannabis-based medicine, cannabis in dentistry, cannabis regulation, clinical applications, ethical considerations, medical cannabis, synthetic cannabinoids

## Abstract

Cannabis and its bioactive compounds, specifically tetrahydrocannabinol and cannabidiol, are rapidly growing in popularity for their therapeutic applications across a variety of medical specialties, including dentistry.

This narrative review aims to explore the current and future applications of cannabinoids in dentistry and the therapeutic potential, problems, and ethical issues.

Cannabinoids possess analgesic, anti-inflammatory, anxiolytic, and neuroprotective properties that may be beneficial in the treatment of orofacial neuropathic pain, temporomandibular joint disorders, myofascial pain dysfunction syndrome, bruxism, and obstructive sleep apnea. Despite their potential benefits, the integration of cannabinoids into dental practice is limited by the absence of standardized treatment regimens, insufficient high-quality clinical evidence, safety concerns, drug-drug interactions, and ambiguously defined legal frameworks. To this, side effects such as xerostomia, increased periodontal disease susceptibility, and deranged surgical and restorative wound healing further complicate clinical use. The recent surge of interest in cannabis-based herbal medications has also brought about uncertainty among patients and dentists regarding their effect on dental health. Ethical concerns such as informed consent, pediatric use, and routes of administration further emphasize the need for careful evaluation.

In the pursuit of safe, effective, and evidence-based use of cannabinoid medicines in dentistry, high-quality clinical trials, targeted professional education, and focused regulatory guidance are needed urgently.

## Introduction and background

Historical background of cannabis use

Cannabis has been medically used for over 5,000 years. Its earliest known use was in ancient China around 2700 BC where it was medicinally used for pain and gastrointestinal ailments, as noted in the Shennong Ben Cao Jing [1]. Cannabis was a prominent drug in ayurvedic medicine in the forms of bhang and ganja, which were valued for their sedative, analgesic, and anti-inflammatory properties [2], and also acquired spiritual significance in texts like the Atharvaveda (around 1400 BC) [3]. Cannabis entered Western medicine during the 19th century. Irish physician William O'Shaughnessy, employed in colonial India, revealed its clinical utility for treating neuralgia, spasms, and toothache [4]. Cannabis tinctures became listed in the British and American pharmacopoeias. Mounting concern about abuse and socio-political issues led to increasing control during the first half of the 20th century, with the final U.S. Marihuana Tax Act of 1937, which effectively ended its medicinal use [5].

Scientific interest resurfaced in the early 2000s with the discovery of the endocannabinoid system (ECS) and the medicinal value of cannabinoids like tetrahydrocannabinol (THC) and cannabidiol (CBD) [6]. The discoveries that followed have sparked research into their therapeutic application, including dentistry, due to their analgesic, anti-inflammatory, and anxiolytic properties [7]. Despite this historical background and the new evidence, the application of cannabis in dental therapeutics remains underappreciated. There is a pressing need to assess its potential applications, maximize existing limitations, and determine its place in dentistry through evidence-based research. This narrative review aims to address this gap by summarizing the therapeutic potential, limitations, and ethical considerations associated with the application of cannabinoids in dentistry.

**Figure 1:**
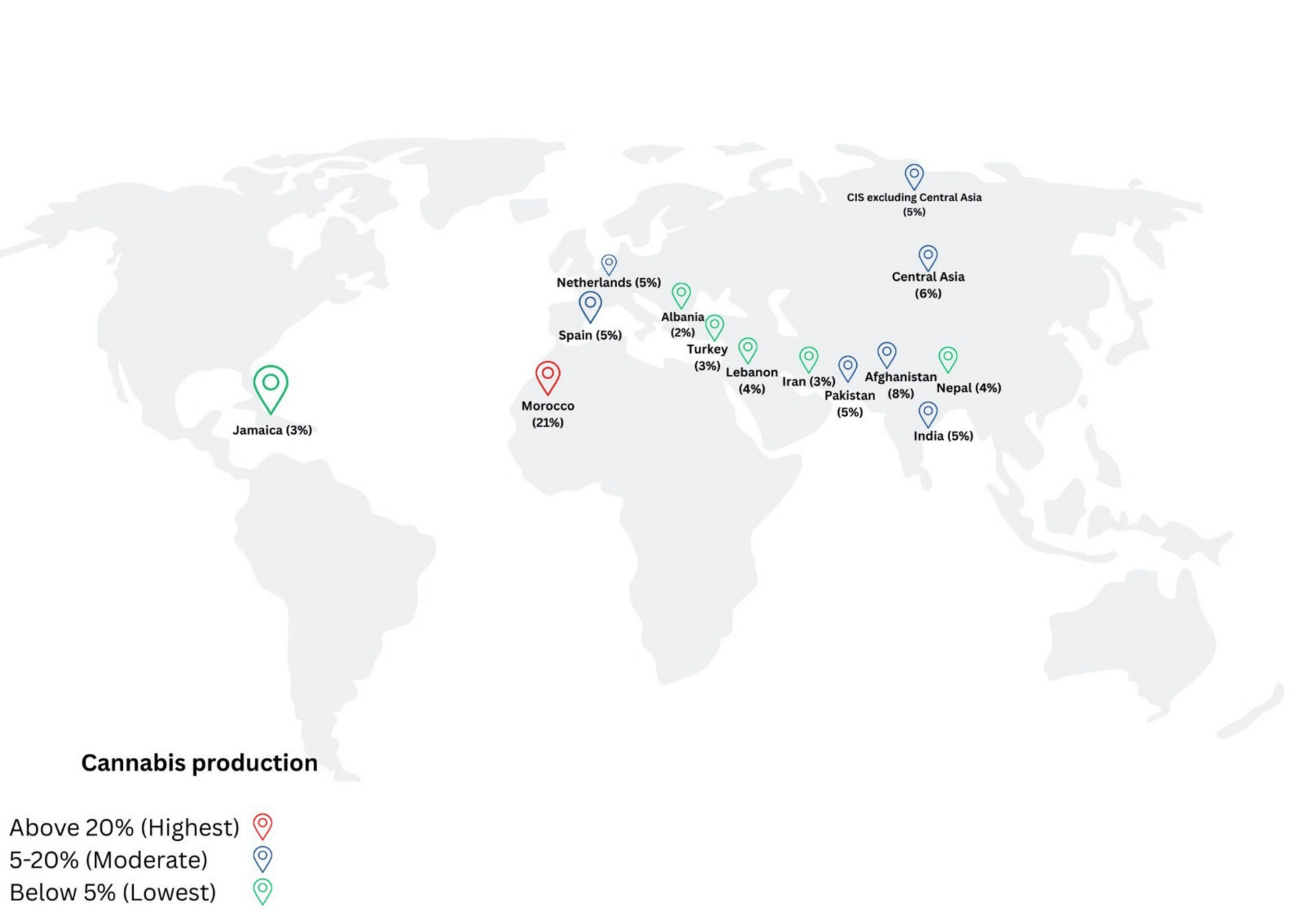
Worldwide Production of Cannabis Image credits: Dr. Shreyas Neelkanthan and Dr. Laresh N. Mistry.

A color-coded world map representing cannabis production levels across various countries and regions is presented in Figure 1. Red markers indicate countries with the highest production (above 20%), blue markers represent moderate production (5-20%), and green markers signify the lowest production (below 5%) [8].

## Review

Pharmacological action of cannabis

Marijuana mainly acts through the modulation of the ECS, a neuromodulatory system responsible for the regulation of pain, mood, appetite, memory, immune response, and inflammation [9]. The ECS is made up of two main G-protein-coupled receptors: CB1, which is mainly found in the central nervous system, such as the hippocampus, basal ganglia, and spinal cord, and CB2, which is widely expressed in immune cells. While CB1 is responsible for motor control, memory, and pain modulation, CB2 has anti-inflammatory and immunomodulatory actions [10]. The endocannabinoids anandamide and 2-arachidonoylglycerol activate these receptors and are broken down by the enzymes fatty acid amide hydrolase (FAAH) and monoacylglycerol lipase (MAGL), respectively [11].

Phytocannabinoids from cannabis, primarily THC and CBD, also act on the ECS. THC is a partial agonist at CB1 and CB2 receptors, having analgesic, antiemetic, and muscle-relaxant effects but being most accountable for cannabis's psychoactivity [12]. In contrast, CBD has low direct affinity for CB receptors but indirectly modulates the ECS by suppressing FAAH and binding to other molecular sites like TRPV1, 5-HT1A, and PPAR-γ, providing therapeutic value with minimal psychoactivity [13]. Such a wide range of interactions is responsible for cannabis's pharmacological diversity in diseases and conditions such as neuropathic and chronic pain, anxiety, inflammation, muscle spasticity, and appetite stimulation, especially in diseases like cancer and HIV/AIDS [14].

But the use of cannabis is not without negative effects. Acute use can cause xerostomia, tachycardia, dizziness, drowsiness, and impaired motor skills. Excessive doses of THC can impair cognition, memory, and judgment, particularly in adolescents or those with psychiatric susceptibility [15]. With chronic consumption, tolerance, dependence, and increased susceptibility to anxiety, depression, or psychosis may occur in susceptible persons [16].

Administration route profoundly affects the pharmacokinetics of cannabis. Smoking and vaping are rapidly acting but have pulmonary risks, while oral administration (edibles, oils, capsules) is slowly acting with long duration and better suited for chronic symptom control [17]. This requires evidence-based, personalized treatment planning to achieve maximum benefits with minimal risks.

**Figure 2:**
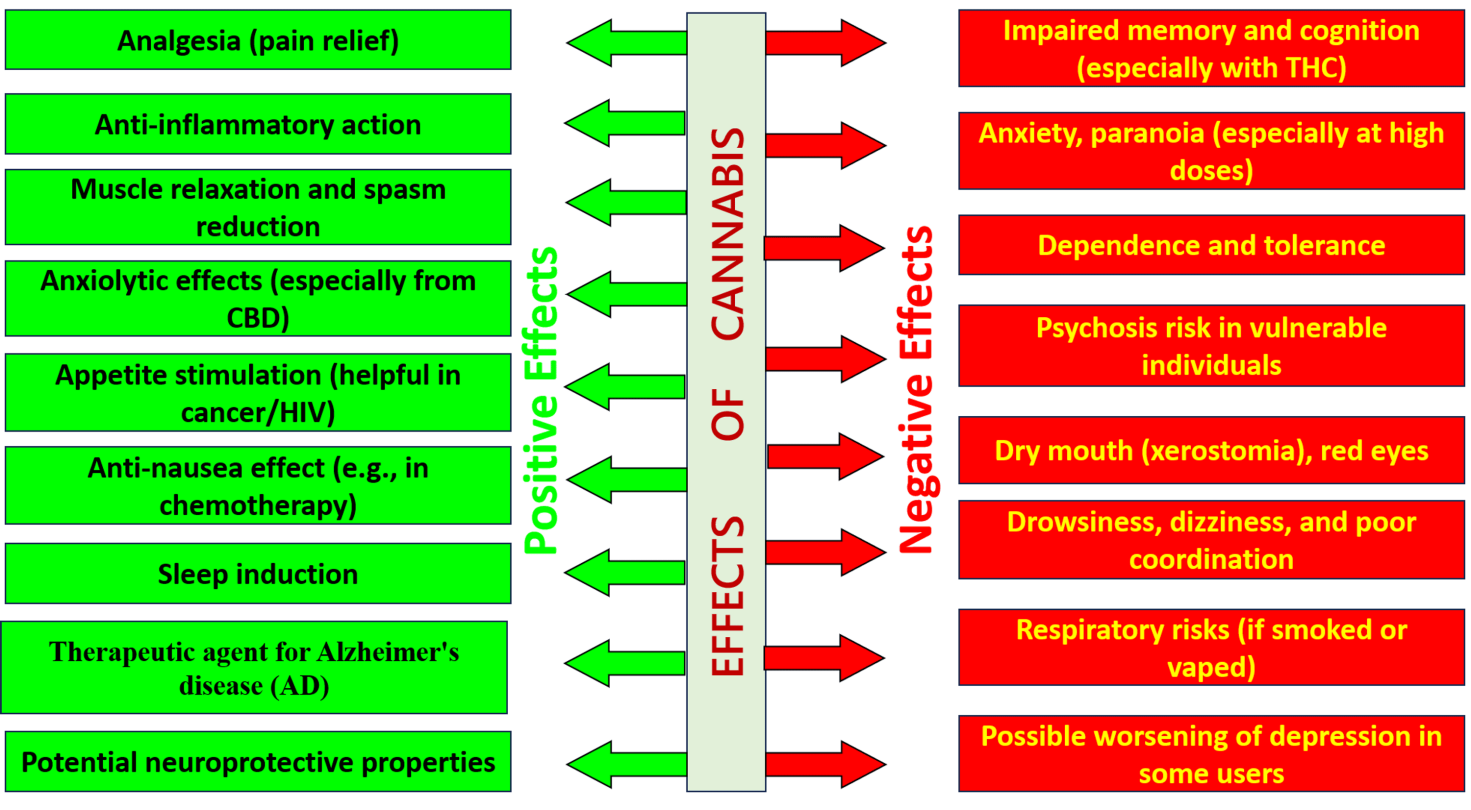
Positive and Negative Effects of Cannabis CBD: cannabidiol; THC: tetrahydrocannabinol. Image credits: Dr. Shreyas Neelkanthan and Dr. Laresh N. Mistry.

The dual pharmacological profile of cannabis displays its therapeutic actions, such as analgesia, anti-inflammation, anxiolysis, neuroprotection, and clinical uses in chronic pain, oncology, and neurodegenerative disease, and adverse effects, such as cognitive impairment, anxiety, dependence, psychosis, xerostomia, dizziness, and respiratory complications. While enlightening, this figure must be viewed critically in clinical situations, highlighting the necessity of individualized risk-benefit consideration, evidence-based selection of routes of administration, extensive patient counseling, and careful monitoring for drug interactions (Figure 2).

Cannabis also affects drug metabolism by controlling cytochrome P450 enzymes, specifically CYP3A4, CYP2C9, and CYP2C19, thus having the potential to change the safety and efficacy of concomitant drugs [18]. THC enhances opioid action, lowering the opioid needs, whereas CBD suppresses the metabolism of drugs such as codeine, tramadol, and nonsteroidal anti-inflammatory drugs (NSAIDs). For example, CBD suppression of CYP2C9 can influence ibuprofen metabolism, enhancing the risk of toxicity or reducing analgesic effect [19]. In addition, simultaneous administration of CYP3A4-inhibiting macrolide or azole antibiotics may increase plasma levels of THC or CBD, potentially increasing sedation or psychotropic effects, particularly among cannabis-naïve patients [20].

A new issue is the changed perception of pain among chronic users of cannabis, which could require increased doses of local anesthetics such as lidocaine or bupivacaine. This might be either via CB1 receptor modulation or changed nociceptive thresholds, with anxiety or hyperalgesia potentially being an added factor, making it crucial to screen for cannabis use in dental pre-procedure evaluation [21]. In addition, inter-individual variations in the metabolism of cannabinoids, commonly associated with polymorphisms in CYP450 enzymes, play a major role in THC and CBD pharmacokinetics. For instance, variants in CYP2C9 may retard the metabolism of THC, intensifying psychoactive effects, while polymorphisms in CYP2C19 can modulate CBD clearance rates. Identification of such pharmacogenomic differences is vital in forecasting patient-specific outcomes, adjusting dosages, and reducing adverse effects. In summary, cannabis's pharmacological effect is complex, with direct and indirect modulation of the ECS, and widespread therapeutic use weighed against significant risks. Care should be taken regarding individual metabolism, genetic heterogeneity, possible drug interactions, and routes of administration for safe and effective clinical application, emphasizing the value of personalized medicine in cannabis-based therapies.

Therapeutic use of cannabis

Cannabis has been an integral part of medicine for centuries, and in the last half century, its clinical uses have been broadened owing to improvements in cannabinoid pharmacology and research into the ECS. It is prescribed legally in nations such as Canada, Germany, Israel, the Netherlands, the UK, and in many US states. Its main indications are chronic and neuropathic pain, particularly in cancer, fibromyalgia, and postoperative contexts. Cannabis also relieves spasticity of multiple sclerosis, with significant improvements from THC: CBD oromucosal sprays. It facilitates cancer chemotherapy-induced nausea and loss of appetite in oncology and HIV/AIDS. Synthetic cannabinoids, such as dronabinol, are licensed for the stimulation of appetite and control of emesis, whereas standardized cannabis-based products are applied to refractory epilepsies, including Dravet and Lennox-Gastaut syndromes [22]. Broader clinical integration, however, requires more intense research, consistent protocols, and explicit guidelines. 

In dentistry, cannabinoids are also being evaluated for their analgesic, anti-inflammatory, anxiolytic, and immunomodulatory properties. THC and CBD are found to have potential in the management of orofacial pain, postoperative pain, neuropathic pain, and periodontitis. The anxiolytic effect of CBD can minimize dental anxiety and enhance compliance, while immunomodulatory activity can assist in wound healing and infection management [23]. In periodontology, cannabinoids might repress inflammatory cytokines through CB2 receptor activation and prevent osteoclast activity to sustain alveolar bone. Preclinically encouraging though data are, systemic and topical dental uses are experimental as of now, with few high-quality human trials [24]. Pediatric treatment is limited due to ethical issues and sparse evidence of safety. While non-psychoactive CBD is being investigated for the treatment of pain and anxiety in autism or cerebral palsy, it involves controlled dosing and monitoring [24]. Cannabinoids can reduce postoperative pain and anxiety and possibly opioid consumption in oral and maxillofacial surgery, but preoperative use of cannabis can affect platelet function, risk of bleeding, and response to anesthetics, so screening before a procedure is necessary [25]. Cannabinoids may also contribute to endodontic pain relief by anti-inflammatory mechanisms, although the absence of clinical trials prevents standard use [26]. Xerostomia, an adverse effect of cannabis, presents difficulties in prosthodontics, impacting denture stability and mucosal health. Cannabinoids have been found in animal studies to affect bone remodeling and thus potentially influence orthodontic tooth movement, though human data are inconclusive [27]. 

THC decreases salivary secretion through CB1 and CB2 receptor action, causing xerostomia, increasing the risk of caries, halitosis, and procedural complications [28]. While promising, cannabinoids in dentistry need to undergo strong clinical trials for therapeutic justification and guideline establishment. New evidence investigates cannabinoids in orofacial conditions. CBD could assist with stress-related bruxism by lowering central nervous tension, although clinical evidence is limited [29]. Synthetic analogs of THC, such as dronabinol, have promise in the management of obstructive sleep apnea through modulation of respiratory control, but evidence is limited [30]. Cannabinoids can be therapeutic for myofascial pain dysfunction syndrome and temporomandibular joint (TMJ) disorders because of their analgesic as well as anti-inflammatory effects, although most evidence is preclinical [31]. Both THC and CBD have potential for the management of neuropathic orofacial pain, such as trigeminal neuralgia, by modulating nociceptive as well as inflammatory mechanisms, possibly acting as alternatives when regular treatment fails, subject to further clinical trials [32]. Although the systemic therapeutic uses of cannabis in conditions like chronic pain, multiple sclerosis, chemotherapy-induced nausea, and appetite stimulation are well established, its uses in dentistry are significantly preclinical. Lack of good-quality randomized controlled trials hinders clinical translation. Further, there is a need for comparative studies comparing cannabinoids with conventional dental analgesics, anxiolytics, and anti-inflammatory agents, but necessary to compare their efficacy, safety, and cost-effectiveness of use in dental practice.

Table 1 presents a summary of essential indications, the purported cannabinoid-based strategies, traditional management modalities, and the current level of supporting evidence. This comparison identifies both the nascent potential of cannabis in the dental practice and the urgent need for additional clinical validation (Table 1).

**Table 1:** Comparative Overview of Cannabis-Based Therapies and Conventional Dental Therapeutics THC: tetrahydrocannabinol; CBD: cannabidiol; NSAIDs: nonsteroidal anti-inflammatory drugs; CPAP: continuous positive airway pressure.

Indication	Cannabinoid-Based Approach	Conventional Dental Therapeutics	Current Evidence Level
Orofacial Pain (Acute/Neuropathic)	THC/CBD—analgesic via endocannabinoid modulation	NSAIDs, opioids, local anesthetics	Preclinical and limited human trials for cannabinoids; strong evidence for conventional agents
Dental Anxiety	CBD—anxiolytic action via 5-HT1A modulation	Benzodiazepines, behavioral therapy	Early clinical studies for CBD; established efficacy for conventional anxiolytics
Periodontitis	CB2 agonists—anti-inflammatory action, osteoclast inhibition	Scaling, root planing, adjunctive antibiotics	Animal studies for cannabinoids; robust evidence for conventional periodontal therapies
Postoperative Pain and Inflammation	THC/CBD—pain relief and inflammation control	NSAIDs, opioids	Preclinical evidence for cannabinoids; established protocols for conventional analgesics
Endodontic Pain	Cannabinoids—anti-inflammatory effects via CB receptors	NSAIDs, analgesics, intracanal medicaments	Limited preclinical data for cannabinoids; conventional therapies clinically validated
Temporomandibular Joint Disorders (TMD)	CBD/THC—modulation of pain and inflammation	NSAIDs, occlusal splints, physiotherapy	Preclinical and anecdotal evidence for cannabinoids; conventional management established
Bruxism (Stress-Related)	CBD—reducing muscle tension and anxiety	Occlusal splints, muscle relaxants	Theoretical basis and early studies for cannabinoids; validated conventional treatments
Obstructive Sleep Apnea	Dronabinol—central respiratory modulation	CPAP, mandibular advancement devices	Limited human data for cannabinoids; CPAP remains standard of care
Neuropathic Orofacial Pain (e.g., Trigeminal Neuralgia)	THC/CBD—nociceptive and inflammatory modulation	Anticonvulsants (e.g., carbamazepine), surgical interventions	Limited clinical trials for cannabinoids; strong evidence for conventional first-line therapies

Side effect profile of cannabis

In addition to its use in various therapies, it may also be used in long-term recreational settings, usually through inhalation, which poses great risks to the oral health of the abuser. THC acts on the CB1 and CB2 receptors to reduce salivary secretion, causing dryness of the mouth, a counteracting measure against dental caries, halitosis, and complications during procedures [28]. Cannabis use is also implicated in the promotion of periodontal disease - the situation may be made worse by immunosuppression, neglect of oral hygiene, or tobacco use as well. Cannabis-containing smoke, in particular, packs carcinogens, such as polycyclic aromatic hydrocarbons, which may be involved in the mutagenesis of the oral mucosa, causing leukoplakia, melanosis, and other pigmented lesions. It is at least conceivable that these changes may have mutagenic concerns, although there is a limited amount of conclusive research on the matter in exclusive cannabis users. Nevertheless, the possibility of mutagenesis calls for a thorough assessment of patients and risk counseling during dental care [25]. 

Cannabis can be responsible for postoperative impairment by inducing changes in immune function and interfering with angiogenesis, fibroblasts, and platelet aggregation. Combined, these processes can lead to unnecessary bleeding and postpone recovery. Long-term use complicates the risk of opportunistic infections, including candidiasis, particularly when the patient is immunocompromised, secondary to the synergetic action of immunosuppression and xerostomia. Furthermore, cannabinoids influence the metabolism pathways of cytochrome P450 enzymes. The most critical conversion of cisoids primarily deals with CYP3A4, CYP2C9, and CYP2C19, the primary enzymes impacting many drugs that are usually prescribed, such as analgesics, antibiotics, and local anesthetics. Hence, it is essential to note the drug interactions in the dental field specifically, especially while negotiating total, safe, individualized doses, and giving a prescription following surgery. Whereas effects of cannabinoids in children, especially in the context of birth defects of the neural tube, were only recently studied conservatively for the treatment of epilepsy, anxiety, and cerebral palsy, there are built-in ethical issues, such as the sparse safety information and the issue of neurodevelopmental toxicities. It remains controversial to treat children's anxiety with cannabinoids, requiring excellent dosing, informed consent, and careful observation in clinical settings. Given the ethical complications associated with cannabis use in children's oral health, the use of cannabis in pediatric dentistry should continue to be as limited as possible and follow evidence-based indications. Screening for cannabis use during the initial dental assessments is an important process of individualizing care, limiting procedural risk, and ensuring informed consent.

Patient's and caregiver's perspective

Societal attitudes around cannabis are evolving, particularly regarding its use as a medicine. Contributing factors include its legalization for medical use, increased public awareness due to campaigns, and patients finding it beneficial for their own conditions, such as chronic pain, anxiety, post-traumatic stress disorder, and symptoms from chemotherapy. Many patients describe cannabis as a natural alternative to pharmaceuticals, and while they acknowledge the lack of scientific evidence, they assert that it improves their quality of life with better sleep, mood, and appetite [33]. Despite finding it beneficial, it is worth mentioning that patients may have limited awareness of potential implications of the use of cannabis on oral health, including xerostomia, delay in wound healing, and interactions with local anesthetics. There are also communication barriers for patients around cannabis use, which include stigma, concerns about legality, and providers assuming patients are not interested in disclosing information about their cannabis use to their dentist. In a survey conducted, it was found that only 34% were comfortable discussing cannabis use with their dentist. Additionally, it is possible that dentists may not inquire about cannabis use due to time constraints, lack of training, and confusion about legality. Finding ways to bridge these gaps is important and may include stigma-free ways of communication and educational resources to promote safer care for patients.

As the popularity of medical cannabis continues to grow, many clinicians are recognizing its potential utility in dentistry. While numerous studies demonstrate that many dental professionals acknowledge its potential as a treatment for pain, anxiety, and inflammation, and acknowledge it as a potential adjunct or alternative to opioids [34,35], its use clinically is still limited. A cross-sectional study of dentists found that over 60% of dentists surveyed acknowledged knowledge deficits about cannabis pharmacology and its medicolegal implications. Additionally, legal uncertainties and concerns contribute to hesitance among practitioners due to unclear patient rights and responsibilities when advising patients on cannabis use [36]. Similarly, a lack of reliable, evidence-based protocols related to dosing, contraindications, and perioperative management creates similar barriers to clinical confidence. Furthermore, the lack of training on the discussion of potential oral complications, such as xerostomia, drug interactions, and healing limitations, reduces the clinician's ability to adequately educate their patients. Providing cannabis-related teaching in dental curricula and continuing education could help address knowledge deficits and empower clinicians to counsel patients appropriately. Legal uncertainty makes its use even more problematic, as dentists are unsure about what rights and responsibilities they may have in making cannabis recommendations [37]. Without established protocols for dosage, contraindications, or perioperative management, many practitioners are hesitant. Moreover, an inability to communicate confidently about possible oral complications, e.g., xerostomia, drug interaction, or delayed healing, forecloses successful patient education [38].

Policy considerations for use in practice

Cannabinoid therapies are now embraced by mainstream medicine in more standardized environments with controls and regulatory systems. Cannabinoids have known anti-inflammatory, analgesic, and antimicrobial potentials, meaning they will provide great value to dental connections, especially in providing effective management of pain, inflammation, and anxiety. Unfortunately, with no guidelines for dental practice, or clinical protocols or regulatory directives, the move to safe, reliable, and judicious incorporation into practice remains stunted [39]. It is likely that the lack of dental-specific regulations has fueled off-label use, poor communication between patient and practitioner about symptoms and treatment options, and increased risk of adverse effects and drug interactions. Large-scale clinical studies, standards of practice, and education of clinician-specific use all merit directions for use and purpose-driven uses with evidence. Artificial intelligence (AI) is emerging as a means of optimizing care in healthcare. AI applications have the potential to advance cannabinoid therapy by analyzing patient-specific details (e.g., medical history, symptoms, genetic details) to develop predictions for needed treatment durations and responses. AI-fueled applications may provide practitioners with cannabinoid-specific dosing recommendations, formulations, and pre-emptively recognize potential drug interactions to optimize and allow purposeful practice in the dental profession. Unfortunately, to date, in the area of dental applications, AI are all mostly unused technologies due to piecemeal integration of policies and standardized plans of action between the dental profession and other related regulated professions. 

Coordination between dental and medical regulators is necessary for the responsible adoption of cannabis-based therapies for oral pain, inflammation, dental anxiety, and periodontitis [40], and education is key to that process. Adding cannabis pharmacology, therapeutic indications, legalities, and safety considerations to dental and medical education will be the heart of ensuring quality care. The greater push is educating students early in their learning about cannabis. As students are exposed to purposeful research involving cannabis use, they may be more innovative as they apply it to dental-specific settings such as CBD-based adjunctive therapies for periodontal treatment or anxiety management. From a regulatory perspective, ethical cannabis use will also require strong limitations on accessibility, pricing, and prescribing practices. The Canadian models, and a few select US States where medical cannabis is regulated for therapeutic use, limit dispensing to licensed government outlets, with dispensers adhering to rigorous ethical standards in dispensing practices. Pricing should be structured to ensure novel medications are made fully accessible to all socioeconomic populations. Access to prescriptions is limited unless there is clearly a clinical advantage to cannabis-based therapy over conventional therapies. Considerations for labeling, packaging, and dispensing should also be informed by further efforts in regulation to eliminate stigma and recreational use while requiring standardized labeling and packaging, and secure dispensing regulations, such as collaborative regulations on prescribing and dispensing by dentists and pharmacies. Similarly, professional associations such as the American Dental Association (ADA) and the FDI World Dental Federation (FDI) can establish evidence-based regulatory working groups on cannabis use in dentistry. The regulatory process advances certain patient protections while promoting an environment of research-based innovation. Establishing and maintaining a specific regulatory supply chain, from producer to consumer, is essential for patient benefit and limiting the potential for misuse.

Ethical implications, limitations, and audit

In dental practice, ethical use of cannabis means that clinicians have openly and fully informed and discussed possible benefits as well as risks and limitations [38]. Vulnerable populations, specifically pediatric patients, must be given special consideration, as there is evidence that CBD can help manage anxiety and pain from neurological disorders in children, even if clinical evidence is limited [39]. Also, although oral administrations are preferable due to a better safety profile and longer therapeutic effect [40], inhalation still has its risks, and many parts of the oral cavity are at risk for significant mounted potential issues, such as xerostomia, periodontal disease, and irritation to the mucosa [28]. Topical uses remain investigational and require validation through controlled studies [41]. The ethical use of cannabis in practice should consider ethical principles beyond informed consent, the condition of distributive justice, which ensures equity in access to cannabis regardless of the socioeconomic condition of the patient, and the consideration of risk and misuse, emphasizing appropriate training and a standardized clinical protocol. It is important that any ethical obligation in practice also respects and upholds the sociological and legal context surrounding cannabis. Systematic audits are one method that will maintain safety and ethical cannabis use within dentistry. An audit system may take into consideration regular reviews of cannabis prescriptions by ethics committees or quality assurance committees within an organization while documenting the indications for use, quantity, route of administration for cannabis products, and patient responses. An audit process should know whether guidelines from organizations were followed, patterns of misuse and overprescribing should be examined, and recommendations and corrective actions should be developed where appropriate. Auditing creates visibility and accountability and is intended to ensure patient safety and support public acceptance for cannabis-based therapies [42].

There are significant limitations to providing cannabis as part of routine dental care, notwithstanding strong support of evidence. For example, there will be no standardized dosing guidelines or routes of administration, or clinical indications. Social and medical considerations of THC would need strong consideration, considering the psychoactive side effects such as dizziness, cognitive impairment, and impairment of judgment, particularly in vulnerable populations. The dental management is further complicated due to oral health-specific adverse effects such as xerostomia, increased risk of caries, mucosal irritation, and delayed healing. In addition, there is a potential for drug interactions between local anesthetics, antibiotics, and analgesics, leading to the need for monitoring and personalized risk evaluation [43]. 

Legal and regulatory gray areas still inhibit large-scale acceptance. Several dental providers remain unsure of their legal obligations and scope of practice, especially with the absence of formal cannabis education in dental school curricula. Moving forward, these obstacles will need to be addressed with rigorous clinical research, standardized treatment protocols, and the addition of cannabis training to formal education. Ethical practice should always consider patient-centered ethical principles of informed consent, distributive justice, equitable access, and honest communication between patients and providers [43].

## Conclusions

The utilization of cannabis in dental practice is a developing field with much potential, given the many analgesic, anti-inflammatory, anxiolytic, and immunomodulatory properties of cannabis. The use of cannabis in medicine is not new; its use has roots as far back as ancient China and India, and is experiencing a modern renaissance in today’s scientific research. While it is not foolproof as a therapeutic option in dental and other medical conditions, the discovery of the ECS has opened the door to explore its potential to manage a variety of orofacial conditions related to chronic pain, bruxism, TMJ disorders, etc.

There is no doubt that the potential of cannabis in the oral environment must be cautiously applied to ensure patient safety and the efficacy of care delivered to patients. To safely implement cannabis in a clinical capacity, evidence-based protocols for oral clinicians, sound regulatory policies, and strong monitoring of prescription-controlled substances are needed. AI will serve as an added tool by offering patient-centric treatment plans based on proven data, limiting unnecessary risks associated with cannabis use in dental treatment. In the future, we need well-designed human trials to examine the efficacy and safety of cannabis-based therapies for dental recommended use. For doctors of dental medicine, establishing dental-focused guidelines for cannabinoid use, regulated legal parameters, and meaningful education in cannabis and oral health during undergraduate dental education will be an important next stage.

This process will allow dental practitioners to better prepare for the ethical, informed, and responsible infusion of cannabis into patient care. With ongoing research, inter-professional collaboration, and a better understanding of state and federal regulations, cannabis could potentially be utilized as a valuable adjunct to dental therapeutics when used thoughtfully, judiciously, and always in the best interests of patients.
